# Management and outcomes of thoracic sarcomas - a collaboration between Orthopaedic Oncology and cardiothoracic surgery: seven-year clinical data from a tertiary referral centre

**DOI:** 10.1186/s13019-025-03341-w

**Published:** 2025-01-27

**Authors:** Zaid Ahmed Shamsi, Vlad Paraoan, Chang Kim, Sarah Raihanah Saifuddin, Thomas D A Cosker, Duncan Whitwell, Christopher L M H Gibbons, Dionisios Stavroulias, Francesco DiChiara

**Affiliations:** 1 Nuffield Orthopaedic Centre, OUH, Oxford, UK; 2 UHCW NHS Trust, Coventry, UK; 3 University Hospitals of North Midlands NHS Trust, Stoke-On-Trent, UK; 4 John Radcliffe Hospital, Oxford, UK; 5 Director of Surgical Anatomy, University of Oxford, Oxford, United Kingdom

**Keywords:** Sarcoma, Thorax, Thoracic sarcoma, Cardiothoracic surgery, Orthopaedic oncology

## Abstract

**Introduction:**

Sarcomas are rare cancers originating from mesenchymal tissues, manifesting in diverse anatomical locations, but notably in connective tissue, muscles and the skeleton. Thoracic sarcomas present a unique diagnostic and surgical challenge attributable to their rarity and pathoanatomy. Standard practice currently comprises wide surgical excision, often accompanied by adjuvant chemotherapy and/or radiotherapy. This approach necessitates a multidisciplinary team, ideally in specialised cancer centres. The Oxford Bone and Soft Tissue Tumour Service is one such centre, and routinely treats such cancers through collaboration between orthopaedic oncology and cardiothoracic surgeons, as well as members of the wider MDT. This study reports the current management and outcomes of primary thoracic sarcoma patients at the Oxford Sarcoma Service over a seven-year period.

**Objectives:**

Given the rarity of thoracic sarcomas, and their associated diagnostic and management complexities, our aim is to report on the treatment strategies and outcomes of primary thoracic sarcoma patients treated at the Oxford Sarcoma Service from 2017 to 2023.

**Methods:**

Data pertaining to all thoracic sarcoma cases discussed in multidisciplinary meetings at the Oxford tertiary centre from 2017 to 2023 were retrieved from the local electronic database. These were analysed using appropriate statistical tests to determine significance of the various observations made.

**Results:**

Of 113 identified cases, chondrosarcoma emerged as the most prevalent histological subtype among 22 distinct varieties. 58% of cases exhibited high-grade features. 32 sarcoma-related deaths occurred, with a mean time from diagnosis to death of 23.16 months. A notable association was observed between high-grade sarcomas and mortality (*p* = 0.0280). Surgical resection was performed in 77 cases, with 49% of these undergoing surgical resection alone i.e. the patient received no radio- or chemotherapy. Both surgical intervention (*p* < 0.0001) and clear margins (*p* = 0.0051) were significantly linked to improved survival. Local recurrence was noted in 28.6% of the 77 surgical cases, and predominantly in the high-grade sarcomas (81.8%). However, no statistical association was found between recurrence and margin status in our data.

**Conclusion:**

Our results indicate that primary resection remains the cornerstone of thoracic sarcoma treatment, representing the single strongest independent factor for survival in treatable cases. Variability in outcomes and overall survival likely stems from factors such as histological diversity, predominance of high-grade sarcomas, and wide age range at diagnosis. Ongoing prospective database update and collaborative efforts across centres would further clarify prognoses and recommendations for specific tumours, based on observational data.

## Introduction

**Fig. 1:**
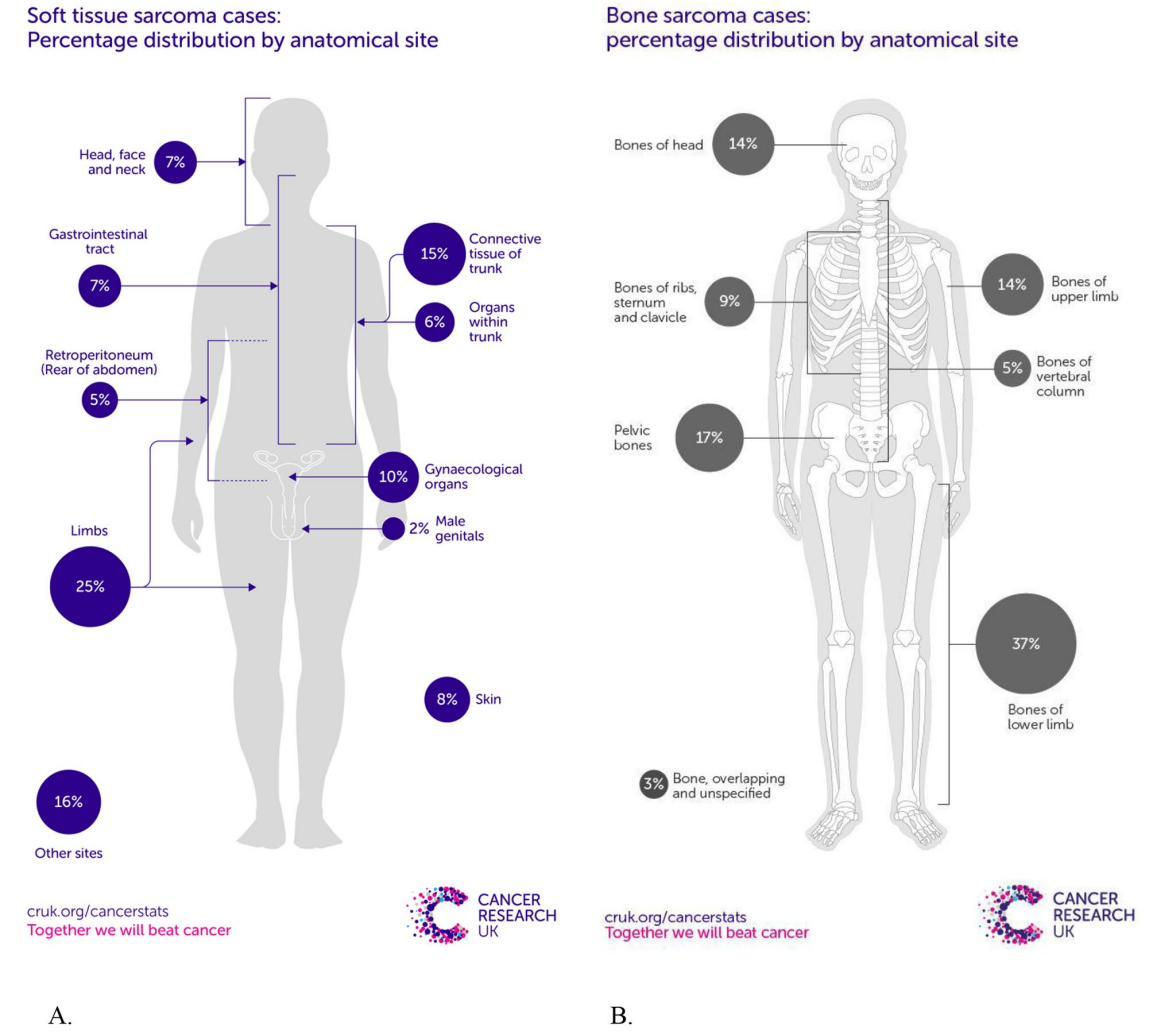
Sarcoma percentage distribution by Anatomical site. **A**: Soft tissue; **B**: Bone

The global incidence of sarcoma is estimated to be 15–20 cases per million population per year [1]. In England, there are c. 4,300 newly diagnosed soft tissue sarcoma cases in England every year, equating to 12 per day. The incidence is highest between the ages of 80 to 84 years. There has been an increase in the incidence of soft tissue sarcoma by 5% in recent years. There are more than 600 bone sarcomas diagnosed in UK each year, accounting for < 1% of all cancer diagnoses [2]. 21% of soft tissue sarcomas are found in the trunk (thorax and abdomen) while 9% of bone sarcomas are found in the thorax (Fig. 1). Sarcomas typically arise de novo, as opposed to an existing pre-malignant lesion – that is to say they are believed to develop spontaneously and completely, contrasting with oesaphageal cancer from Barrett’s oesophagus, for example. Thoracic sarcomas are extremely rare, and their diagnosis is typically established only after excluding primary lung cancers or metastasis from distant primary cancers, including other sarcomas [3–5].

Considering primary soft tissue sarcomas of the thorax, these are most commonly angiosarcoma, leiomyosarcoma, rhabdomyosarcoma, or a sarcomatoid variant of mesothelioma, and mainly originate in pleura, lung and the mediastinum [6], . Primary bone sarcoma on the other hand is most frequently chondrosarcoma, the most common anatomical location being the anterior ribs or sternum. They account for 7-19% of all chondrosarcomas [7].

The low incidence of thoracic sarcoma underpins the lack of evidence-based guidelines, and indeed health-related quality of life measures, for the management of such cancers [8–10]. They are most commonly treated with similar strategies to extremity soft tissue sarcomas, for which more established guidelines exist [10]. Surgery remains the primary modality of treatment of primary thoracic sarcoma. The role of radiation and chemotherapy has yet to be fully established [11, 12], but nonetheless they are still used as an adjunct to surgery, either in preceding or ensuing formats [12]. Overall, the main challenge for surgeons remains achieving a surgical resection with adequate margins, which is often extremely challenging due to the anatomic location of such tumours and their proximity to vital structures. Therefore, multidisciplinary collaboration is critical for the diagnosis of these cancers and subsequent planning of management [13].

The Oxford Bone and Soft Tissue Sarcoma Service is one of the five tertiary sarcoma referral centres in the UK, and the only one that routinely integrates other surgical specialties, namely cardiothoracic, gynae-oncology, head and neck, paediatric, abdominal surgery etc. in diagnosis and management of sarcomas in all bodily compartments.

This paper presents the management and subsequent outcomes of primary thoracic sarcomas presenting to the Oxford Sarcoma Service over a seven-year period (2017–2023).

## Methods

Data on all thoracic sarcoma patients discussed at the Oxford tertiary centre multidisciplinary meetings over a seven-year period (2017 to 2023) were retrieved from electronic patient records. Inclusion criteria were patients with primary thoracic sarcomas, ICD-10 code C49.3, defined as malignant neoplasm: Connective and soft tissue of thorax, between the clavicles/lung apices and the diaphragm.

The collected data encompassed patient demographics (age at diagnosis, sex), histological origin and grade, soft tissue or bone involvement, anatomical location, initial treatment, marginal status, recurrence, treatment modality for recurrence, presence of metastases.

at diagnosis, sarcoma-related mortality, and mean survival.

Histological grading of specimens was performed by specialised sarcoma histopathologists using the American Joint Committee on Cancer (AJCC) system. In this study, grade 1 tumours were classified as low-grade, while grade 2 or higher were classified as high-grade.

All data were consolidated in a central database, to permit subsequent statistical analysis. The Shapiro–Wilk test was used to assess normality. Non-parametric methods were employed in cases of non-normal distribution. Data were grouped by marginal status, histological grade, and treatment. Continuous variables were compared using the non-paired t-test or ANOVA, while dichotomous variables were compared using Fisher’s exact test. Survival estimates were generated using the Kaplan–Meier method and analysed with Log-rank test. Overall survival was defined as the time from diagnosis to death from any cause, measured in months, with appropriate censoring for patients who had not yet reached the relevant follow-up periods.

Statistical significance was set at *p* < 0.05. Analyses and figure generation were undertaken in GraphPad Prism 8.

## Results

113 patients met inclusion criteria. There were 67 male and 46 female patients. Mean age at diagnosis was 54.42 years (SD: 19.41 years, Range 3–87 years). Demographic data of all patients is shown in Table 1. At the time of study, 32 patients (28% of the studied population) had succumbed to their disease, with a mean time of 23.16 months from the date of diagnosis to death. 81 of 113 patients had soft tissue sarcomas, most commonly arising from the chest wall (*n* = 21, 18.6%). 32 patients had primary bone sarcoma, with the rib cage being the most common site of origin (*n* = 23). 22 different histological subtypes were recorded with chondrosarcoma being the most common (26%, *n* = 29) followed by spindle cell sarcoma (10%, *n* = 11). Table 1 illustrates the anatomical distribution and histological subtypes.

**Table 1:** Demographics

Category	Details	Frequency	Percentage (%)
**Total Patients**		113	100.0
**Age Range**		3–87 years	-
**Gender**	Male	67	~ 60.0
	Female	46	~ 40.0
**Primary Tumor Location**	Chest Wall	21	18.6
	Rib	23	~ 20
	Lung	15	13.3
	Mediastinum	10	8.8
	Back & Pleura	7 each	6.2 each
**Histological Subtypes**	Chondrosarcoma	29	21.2
	Spindle Cell Sarcoma	11	25.7
	Undifferentiated Pleomorphic Sarcoma (UPS)	7	6.2
	Myxofibrosarcoma	7	6.2
	Ewing’s Sarcoma	7	6.2
	Liposarcoma	7	6.2
	Other Rare Subtypes	Remaining cases	~ 43.4
**Tumor Grade**	High Grade	66	58.4
	Low Grade	23	20.5
	Unknown Grade	24	21.2
**Clinical Outcomes**	Recurrence	22	19.4
	Metastases	25	22.1
	Sarcoma-Related Mortality	32	28.3

**Fig. 2:**
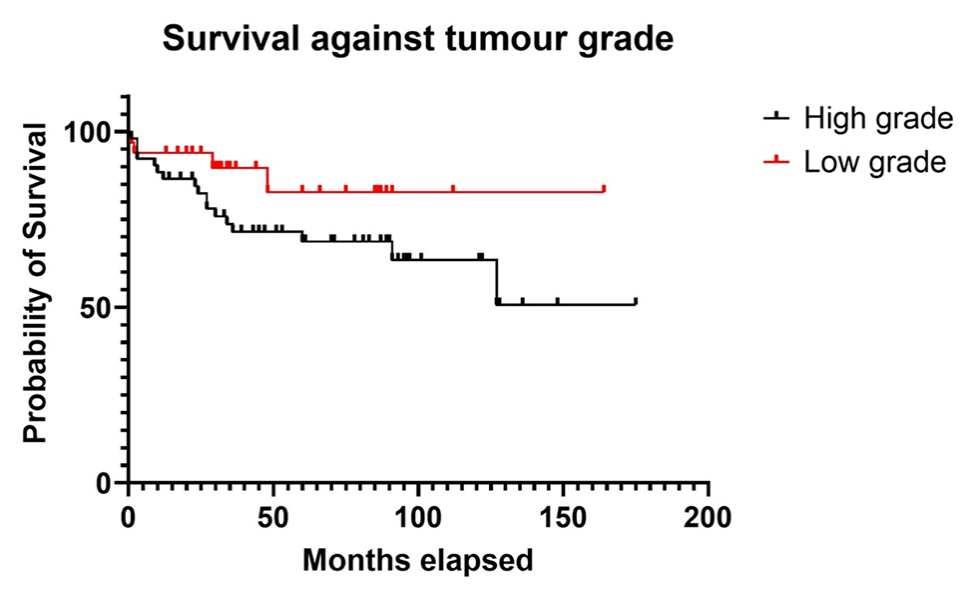
Survival by histological grade

66 (58.4%) patients were classified as high-grade cases, while 24 (21.2%) patients were low-grade. 23 (20.3%) patients had an indeterminate grade. Of the 32 patients who succumbed to their disease, 20 had high-grade tumours, compared to 4 with low-grade tumours (the rest being indeterminate). Table 2 summarises the data according to histological grade, with Fig. 2 confirming survival.

**Table 2:** Analysis by histological grade

	Low grade	High grade	
**n**	25	44	
**Age**,** mean (SD)**	46.8 (14.7)	53.9 (19.0)	*p* = 0.1122
**Non-surgical therapy**			*p* = 0.0110*†
**None**	22	12	
**Chemotherapy**	1	9	
**Radiotherapy**	2	18	
**Chemo-radiotherapy**	0	3	
**Margins**			*p* = 0.3883
**Clear**	17	30	
**Marginal**	2	4	
**Involved**	0	6	
**Recurrence**	2	18	*p* = 0.0280*
**Sarcoma-related Mortality**	1	10	*p* = 0.0925

### Treatment

77 out of 113 patients (68.1%) patients underwent wide surgical resection. Of these, the final histological grading was determined as high-grade in 44, low-grade in 25, and indeterminate in 8.

38 surgical patients did not receive any form of adjuvant treatment; 11 received chemotherapy alone; 20 received radiotherapy alone; 6 patients had chemo-radiotherapy.

Table 3 summarises data with respect to surgical intervention and adjuvant therapy. We found a statistically significant age difference between the two groups. Surgical patients were found to be younger (mean age 50.9) than patients who did not undergo surgery (mean age 61.9) with a p-value of 0.0046. As expected, surgery was associated with a lower rate of metastatic disease at presentation (*p* = 0.0103).

**Table 3:** Analysis by surgical intervention

	Non-surgical	Surgical	
**n**	36	77	
**Age**,** mean (SD)**	61.9 (19.0)	50.9 (18.6)	*p* = 0.0046**
**Grade**			*p* = 0.3826
**Low**	9	25	
**High**	12	44	
**Metastases**	13	7	*p* = 0.0103*
**Non-surgical therapy**			*p* = 0.0039**†
**None**	23	38	
**Chemotherapy**	4	11	
**Radiotherapy**	3	20	
**Chemo-radiotherapy**	5	6	
**Sarcoma-related Mortality**	20	12	*p* = 0.0035**

**Fig. 3:**
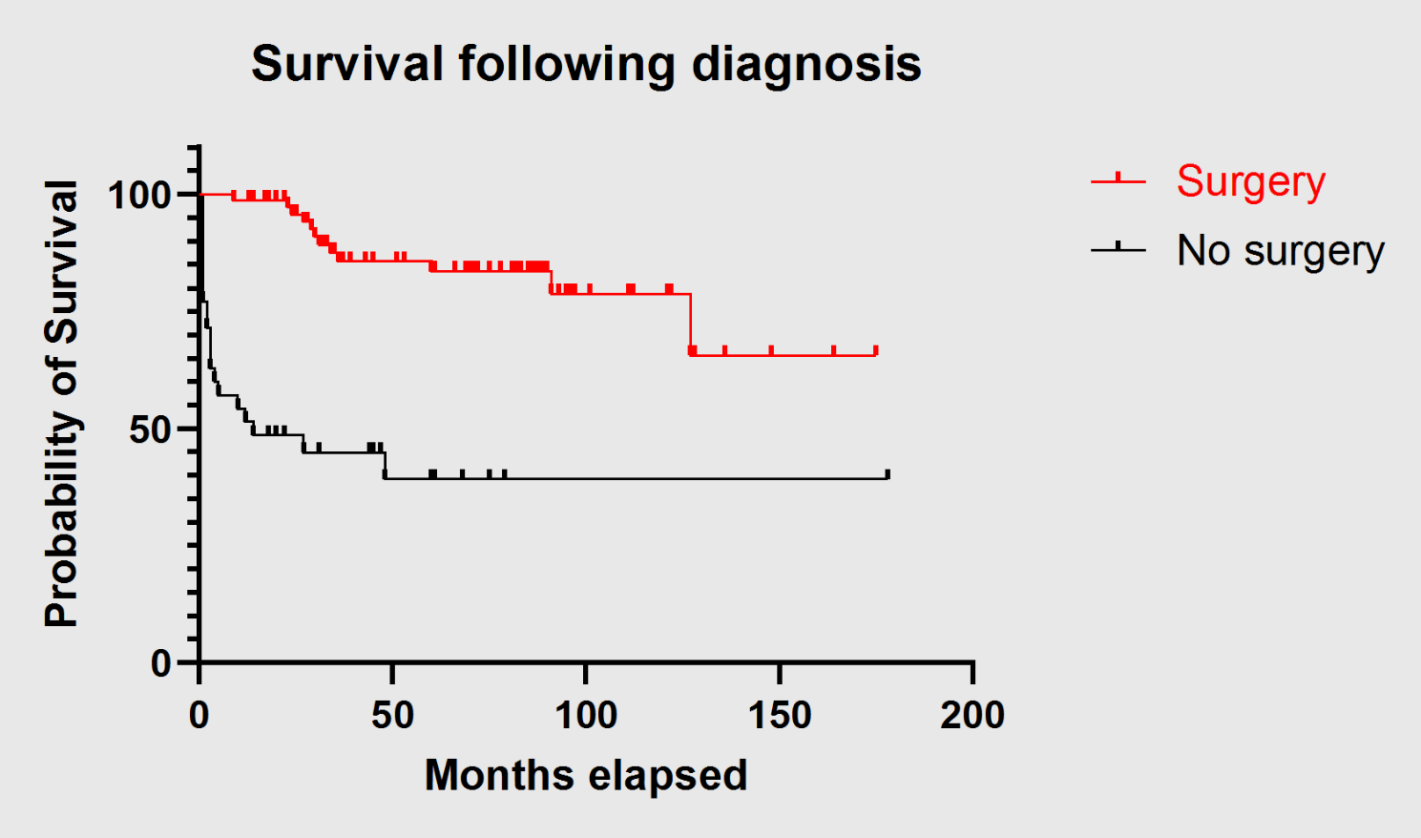
Survival following surgical intervention

Surgical intervention also had a statistically significant benefit with respect to survival (*p* = 0.0035). No other statistically significant association was found in the two groups. Figure 3 shows the survival according to surgical intervention.

### Marginal status

**Fig. 4:**
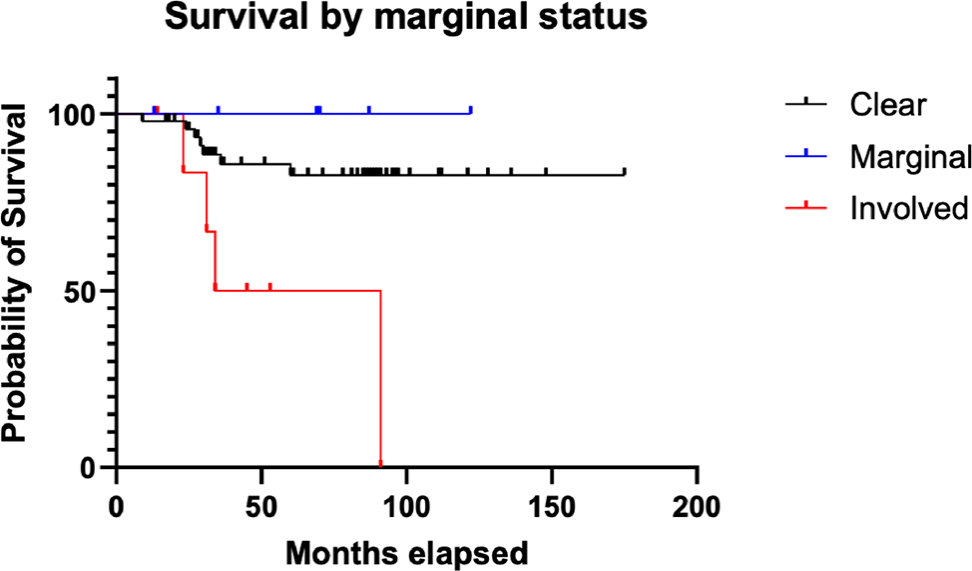
Survival by marginal status

We found no association between marginal status and histological grade of the patients who underwent surgery. 50 out of 77 (65%) surgical patients had clear margins, 6 (8%) had marginal margins, 7 (9%) had involved margins, and 14 (18%) had unclear margins. Improved survival outcome was again demonstrated with clear and marginal margins (*p* = 0.051). Table 4 summarises these findings. Figure 4 shows survival by marginal status.

**Table 4:** Analysis by marginal status

Margin Status	Number of Patients (*n*)	Percentage (%)
Clear Margins	50	65%
Marginal Margins	6	8%
Involved Margins	7	9%
Unclear Margins	14	18%

Note: Improved survival outcome was observed in patients with clear and marginal margins (*p* = 0.051)

### Recurrence

22 (28.6%) patients had local recurrence at the end of our study. 18 (81.8%) of these occurred in high-grade cases, while only 2 patients with low-grade sarcoma had local recurrence (Table 2). 40.9% of patients with high-grade sarcoma had recurrence. This was demonstrated to be statistically significant with a p-value of 0.0280. No association between marginal status and recurrence was demonstrated in this study.

## Discussion

Few studies have been published focusing primarily on the management of thoracic sarcomas. This is because they constitute a small proportion of all sarcomas, which in themselves are rare entities. 5% of all thoracic cancers are primary sarcomas while sarcomas only form 0.2% of all lung cancers [4]. To our knowledge, no treatment guidelines have yet been published [8]. Our study represents one of the largest worldwide series of thoracic sarcomas treated at a single institution using multidisciplinary team working, focusing on the treatment and outcomes of these rare neoplasms.

We had a wide age range in our study (3–87 years) with a mean age of 54.42 at the time of diagnosis. This is in-keeping with previous published data [14–16]. Our male-to-female ratio was comparable to other studies; 59% of our study population were males. Soerensen et al. also reported a similar male-to-female ratio in their study of thoracic sarcomas [14]. However, there are other articles that demonstrate a higher proportion of female patients in their studied population [15, 16].

Our study predominantly consisted of soft tissue sarcoma cases, with the STS-to-bone sarcoma ratio being 2.5:1. Whilst it is frequently observed that STS occur more commonly than bone sarcomas, the reported extent in the literature is markedly variable, with some studies reporting it as high as 4:1 [17]. Most of our sarcomas (47%) originated from the chest wall which is again comparable to most previous studies. We identified 22 different histological subtypes, with the most common being chondrosarcoma (26%) followed by spindle cell sarcoma (10%). This finding echoes what others have also reported: Friesenbichler et al. and Walsh et al. both report the incidence of chondrosarcoma at 22.4% and 29.6%, respectively [18, 19]. Jokerst et al. also report that chondrosarcoma is the most common primary malignant neoplasm of the chest wall. Chest wall tumours comprise 7–19% of all chondrosarcomas with an apparent predilection for the upper 5 ribs [7, 8].

Although surgery remains the mainstay of treatment for localised sarcomas, recent studies suggest that incorporating (neo)adjuvant radiation and/or systemic therapy can potentially improve outcomes [19–21]. Pervaiz et al. published a meta-analysis of 1953 patients showing improved rates of recurrence and overall survival in patients receiving chemotherapy for localised resectable STS [22]. A separate meta-analysis published by Albertsmeier et al. in 2018 demonstrated improved recurrence rates in patients receiving neo-adjuvant or adjuvant radiotherapy [23]. Of course, these treatment modalities are themselves associated with risks of significant complications, hence treatment should be individualised to each patient after careful discussion, again relying upon a multidisciplinary approach. The Oxford Sarcoma Service adheres to these principles, discussing all sarcoma patients in their weekly MDT meeting with the involvement of all relevant surgical specialties, clinical oncologists, MSK radiologists, sarcoma histopathologists and senior sarcoma nurses.

77 of the 113 patients identified underwent surgical resection. 37 of these also had some form of adjunct therapy: 11 received chemotherapy alone, 20 had radiotherapy alone, and 6 had both. Of the 36 patients who did not undergo surgery, 4 received chemotherapy, 3 received radiotherapy, and 5 received both. Surgical intervention was strongly associated with a higher survival rate compared to the non-surgical group (*p* = 0.0035). Surgical candidates were also found to be younger (*p* = 0.0046) and had absent metastatic disease (*p* = 0.0103). Our findings corroborate the improved survival seen with surgical intervention in the wider literature [15, 24–28].

We report an overall survival of 72% with a total of 32 sarcoma-related deaths at a mean time of 23.16 months from the time of diagnosis. 62.5% of deaths occurred in patients with high-grade sarcoma. This study also shows statistical association between marginal status and overall survival, with clear and marginal margins conferring a clear survival benefit compared to involved margins (*p* = 0.0051). Our findings corroborate other papers, including a recently published study of 121 patients demonstrating survival benefit with a clear margin, and poorer outcomes in cases with high-grade neoplasms [15, 19, 27, 29, 30].

22 patients in our study had local recurrence, accounting for 28.6% of all surgical patients. Notably, 18 (81.8% ) of these were found in high-grade sarcoma patients. Only 2 patients with low-grade sarcoma had recurrence during our study period (*p* = 0.0280). Soerensen et al. reported a similar recurrence of 33%, while Tskukushi et al. reported a lower recurrence of just 11% in their study [15, 31]. This is best explained by the decreased number of high-grade tumours, and the inclusion of borderline tumours in their study. Interestingly, we did not find any statistical association between surgical margins and local recurrence rate (*p* = 0.0509). This is supported by Strauss et al. who could not reveal an effect of metric margin width as a prognostic factor for local recurrence in their retrospective analysis of 210 patients over a 20-year period at a national referral centre [32]. Likewise, Soerensen et al. found no statistically significant difference among patients with clear margins vs. involved margins with respect to local recurrence rates [15].

The limitations of our study include its retrospective design, single-institute data, and the wide variety of histological subtypes each with their own oncological characteristics leading to difficulty in generalising treatment. There is potential for selection and recall bias, given that we analysed past data that was not specifically intended for research purposes. There was also incomplete data, limiting the conclusions we can draw from our analyses. However, our study is the first to present the management and outcome of thoracic sarcomas at a tertiary referral centre where surgical intervention is jointly performed by cardiothoracic surgeons and orthopaedic oncology surgeons. This study will serve as a benchmark for future larger prospective multi-centre studies evaluating strategic approaches in the management of specific thoracic sarcomas. Data collection would be informed by our findings and supplemented with a Delphi process with experienced clinicians treating sarcoma, in order to undertake further subgroup analyses with respect to different histological subtypes, and patient demographics. In this way, we hope to offer a higher level of evidence to the medical community for informing tailored strategies for the treatment of specific tumours in specific patients.

## Conclusion

Our study demonstrates that surgical resection should remain the principal intervention in thoracic sarcoma when possible, owing to the significant survival benefit conferred. Given the broad histological subtypes and presentations of thoracic sarcomas, a multidisciplinary approach to their management is critical. Co-ordination among surgery, radiation, medical oncology, pathology, and radiology teams is necessary to ensure clearer classification and effective management strategies for these rare malignancies. To improve patient survival and outcomes for these rare diseases, it is critical that treatment be delivered at specialised centres. Collaboration between us can only serve to find answers as to how best to treat these cancers at a much needed, faster pace.

## Data Availability

No datasets were generated or analysed during the current study.
